# Pardon My Sobbing: A Qualitative Study on Differentiating Generalised Mood Disorders From Premenstrual Dysphoric Disorder

**DOI:** 10.1192/bjo.2022.235

**Published:** 2022-06-20

**Authors:** Alisha Pradhan

**Affiliations:** Royal Cornwall Hospital, Truro, United Kingdom

## Abstract

**Aims:**

To assess current diagnostic methods used in the United Kingdom which have led to successful diagnoses of PMDD (Premenstrual Dysphoric Disorder).

**Methods:**

Women suffering from PMDD were recruited across the United Kingdom. Participants were interviewed using a semi-structured interview guide about their paths to diagnoses and the impact of receiving a misdiagnosis. Interviews were transcribed and thematically analysed to assess for overarching themes and similarities across participants. The Patient Health Questionnaire-9, Generalised Anxiety Disorder-7 survey, and Premenstrual Symptom Screening Tool were used to assess for each questionnaire's diagnostic ability.

**Results:**

Four women aged 30–50 years all identifying as PMDD patients were interviewed. All four participants received misdiagnoses of major depressive disorder and/or generalised anxiety disorder. The key to achieving a PMDD diagnosis for all four women was awareness of the cyclical nature of their symptoms. All three surveys failed to fulfill all the diagnostic criteria for PMDD, however the Premenstrual Symptom Screening Tool performed the best and elicited the greatest number of symptoms from the population sample.

**Conclusion:**

This research showed the need for patient awareness of PMDD via research, or awareness of the relationship between symptoms and the menstrual cycle, to achieving a diagnosis and receiving adequate treatment. Specialist treatment was also imperative to achieving a formal diagnosis.

